# Disease Specific Autoantibodies in Idiopathic Inflammatory Myopathies

**DOI:** 10.3389/fneur.2019.00438

**Published:** 2019-05-08

**Authors:** Bruno Stuhlmüller, Udo Schneider, José-B. González-González, Eugen Feist

**Affiliations:** ^1^Department of Rheumatology and Clinical Immunology, Charité-Universitätsmedizin, Berlin, Germany; ^2^Labor Berlin-Charité Vivantes GmbH, Berlin, Germany

**Keywords:** myositis, inflammation, autoantibodies, antigens, biomarker

## Abstract

Idiopathic inflammatory myopathies represent still a diagnostic and therapeutic challenge in different disciplines including neurology, rheumatology, and dermatology. In recent years, the spectrum of idiopathic inflammatory myopathies has been significantly extended and the different manifestations were described in more detail leading to new classification criteria. A major breakthrough has also occurred with respect to new biomarkers especially with the characterization of new autoantibody-antigen systems, which can be separated in myositis specific antibodies and myositis associated antibodies. These markers are detectable in approximately 80% of patients and facilitate not only the diagnostic procedures, but provide also important information on stratification of patients with respect to organ involvement, risk of cancer and overall prognosis of disease. Therefore, it is not only of importance to know the significance of these markers and to be familiar with the optimal diagnostic tests, but also with potential limitations in detection. This article focuses mainly on antibodies which are specific for myositis providing an overview on the targeted antigens, the available detection procedures and clinical association. As major tasks for the near future, the need of an international standardization is discussed for detection methods of autoantibodies in idiopathic inflammatory myopathies. Furthermore, additional investigations are required to improve stratification of patients with idiopathic inflammatory myopathies according to their antibody profile with respect to response to different treatment options.

## Introduction

Idiopathic inflammatory myopathies (IIM) represent a heterogeneous group of acquired muscle diseases with so far unclear etiology. The different entities are associated with diverse clinical symptoms ranging from amyopathic to necrotic inflammatory muscle involvement, typical skin and internal organ involvement. In addition to the clinical picture, histological and serological findings are especially supportive in differentiation and stratification of disease (1). Based on this characteristics, IIM can be classified into different major sub-types: (i) polymyositis (PM), (ii) sporadic inclusion body myositis (sIBM), (iii) dermatomyositis (DM), (iv) immune-mediated necrotizing myopathy (IMNM), and (v) overlap syndromes with myositis (2, 3). Within the spectrum of antibodies two classes have been proposed (4), designated as myositis specific antibodies (MSAs) due to their exclusive association with IIM or as myositis-associated antibodies (MAAs) due to their prevalence in different other connective tissue disorders. This article focuses on an overview on so far identified specific autoantibody-antigen systems in IIM. Furthermore, we discuss the available methods, strategies and pitfalls of autoantibody detection in IIM as well as their diagnostic performance and clinical association.

## Characterization of Disease Specific Antigen-Antibody Systems in IIM

As with many other systemic autoimmune diseases, it is unclear so far, whether and how the observed autoantibody formation is directly associated with the pathogenesis of disease or is just an epiphenomenon. However, the striking association between certain autoantibodies with a distinct clinical phenotype, their high disease specificity and their value for stratification and prognosis of disease suggests that they may play a role in disease induction and propagation (5). This section describes in detail the nature and function of so far identified IIM specific antigens. A complete overview on so far identified antigens and the corresponding autoantibodies in adult and in juvenile patients with IIM is given in Table 1.

**Table 1:** Characterization of myositis specific and myositis associated autoantibodies and their respective antigens.

	**Autoantigen name and function**	**Molecular weight in kilodalton**	**Cellular localization**	**Disease and associated manifestations**	**Frequency in adult (A) and juvenile (J) IIM**	Conventional and commercial (^*^) detection systems	**References**
**ANTI-SYNTHETASE SYNDROME (ASS)**							
EJ	Glycyl-tRNA synthetase	79	Cy	ASS with mechanic hands, associated with increased risk of interstitial lung disease	(A) < 5%	ELISA, IP & WB, ^*^IIFT ^*^Strip test	(6)
Jo-1	Histidyl-tRNA synthetase	60	Cy	ASS with interstitial lung involvement, mechanic hands, Raynaud, arthritis	(A) 20–30%	ELISA, ID, IP & WB, ^*^IIFT, ^*^Strip test	(7–10)
OJ	Isoleucyl-tRNA synthetase	145	Cy	ASS with interstitial lung disease, sometimes also found in RA	(A) 3%	ELISA, IP & WB, IF, ^*^IIFT, ^*^Strip test	(6, 11)
Pl-7	Threonyl-tRNA synthetase	83	Cy	ASS with increased risk of interstitial lung disease, Raynaud syndrome,	(A) < 5%	ELISA, ID, IP & WB, ^*^IIFT, ^*^Strip test	(8)
Pl-12	Alanyl-tRNA synthetase	108	Cy	ASS associated in 90% with interstitial lung disease	(A) 2–3%	ELISA, ID, IP & WB, ^*^IIFT, ^*^Strip test	(12)
KS	Asparaginyl-tRNA synthetase	51	Cy	ASS	(A) < 5%	ELISA, IP & WB	(13, 14)
Ha	Thyrosyl-tRNA synthetase	59	Cy	ASS	(A) < 1%	ELISA, IP & WB	(15)
Zo	Phenylalanyl-t-RNA synthetase	52	Mi	ASS frequently with fever	(A) < 1%	ELISA, IP & WB	(15, 16)
**DERMATOMYOSITIS (DM), POLYMYOSITIS (PM) AND IMMUNE MEDIATED NECROTIZING MYOPATHY (IMNM)**							
SRP	Signal recognizing particle	72/52	Cy	IMNM, acute und subacute	(A) 5–10% (J) < 3%	WB, IF, ^*^IIFT ^*^Strip test	(7, 17, 18)
MDA-5 / CADM-140	Melanoma differentiation associated Gene 5	140	Cy	Amyopathic dermatomyositis, mostly in relation with progressive interstitial pneumonia	(A) 50–73% in Asian populations	WB, IF, ^*^IIFT	(19, 20)
Mi-2α and β	Nucleosome deacetylase complex; helicase binding protein	240	Nu	DM mild forms	(A) 15–30% (J) 10–15%	WB, IF, ^*^IIFT, ^*^Strip test	(21, 22)
NXP-2 (MJ)	Nuclear matrix protein 2	140	Nu	DM und juvenile DM/PM, mostly associated with calcinosis, in juvenile DM involved with ischemia and in adult PM with cancer	(A) 18–25% (J) 60%	WB, IF, ^*^IIFT, ^*^Strip test	(23, 24)
SAE1/2	Small ubiquitin like modifying and activating enzyme	90/40	Nu	DM, often also found in patients with amyopathic DM	(A) < 5% (J) < 1%	WB, IF, ^*^IIFT, ^*^Strip test	(23, 25, 26)
TIF-1γ	E3-Ligase, transcriptional factor-1γ hetero complex	155/140	Nu	Cancer associated DM in adults, skin ulcerations in juvenile DM	(A) 13–21% (J) 22–29%	IF, WB, ^*^IIFT, ^*^Strip test	(23, 27)
p200/100 HMGCR (HMG-CoA)	3-hydroxy-3-methylglutaryl-CoA reductase	200/100	ER membrane	IMNM frequently associated with prior statin use	(A) 6-10 %	IP and WB	(28–31)
**MYOSITIS ASSOCIATED ANTIBODIES (MAA)**							
Ku70 / Ku80	DNA-binding protein kinase complex, involved in DNA double strand repair	70/88	Nu	Overlap-Syndrome frequently with pulmonary fibrosis, arthralgia, gastrointestinal involvement,	(A) 7%	WB, IF, ^*^IIFT, ^*^Strip test	(32)
cN-1A	Enzyme, Cytosolic 5′-nucleotidase	43	Nu	High frequency in sporadic inclusion body myositis (sIBM), but also in Sjogren's syndrome, infrequent in other forms of IIM	(A-) s-IBM 33–44%, PM/DM 5%	ELISA, IF, ^*^IIFT, ^*^Strip test	(33–36)
U1-RNP	U1-ribonuclear Protein	70	Nu	Associated with mixed connective tissue diseases (MCTD)	(A) up to 12%	WB, IF	(32, 37)
SS-B (La)	RNA-metabolism and processing of pre-tRNA; viral RNA binding protein, RNA-chaperone	50	Nu	Associated with Sjogren's syndrome and systemic lupus erythematosus (SLE)	(A) 10%	WB, IF	(38, 39)
SS-A (Ro)	E3-ubiquitine ligase	60/52	Nu / Cy	Associated with Sjogren's syndrome and SLE	(A) 50% (J) 10%	WB, IF, ^*^IIFT, ^*^Strip test	(40–42)
PM/Scl	Exo-ribonuclease	100/75	Nu / Cy	Overlap syndrome with myositis and scleroderma	(A) 5%	IF, WB, ^*^IIFT ^*^Strip test	(7, 43)

*ID, Immunodiffusion; IIFT, Indirect Immunofluorescence test; IF, Immunofluorescence; WB, Immunoblotting; IP, Immunoprecipitation; MI, mitochondrium; Cy, cytoplasm; Nu, nucleus; (A), adult IIM; (J), juvenile IIM*.

The best-known autoantigen-autoantibody system in IIM is directed against **transport ribonucleoacid (t-RNA) synthetases** and represents a specific finding in patients with so named anti-synthetase syndrome (ASS). The targeted synthetases catalyze the binding of a specific aminoacid to their t-RNA in the cytoplasm of each eukaryotic cell for transportation to the ribosome and subsequent protein synthesis. At least eight t-RNA synthetases have been identified as autoantigens. Anti-Jo1 antibodies are the most common ones and received their designation after the initials of the index patients (44). It is unclear, why not all t-RNA synthetases are targeted by the immune system in ASS, but only the tRNA synthetase for threonyl (PL-7), alanyl (PL-12), isoleucyl (OJ), glycyl (EJ), asparaginyl (KS), phenylalanyl (Zo), tyrosil (Ha), and finally the histidyl synthetase (Jo-1). Even more of interest is the question, whether the antibodies can interfere with the function of the respective t-RNA synthetase as it has been shown for anti-Jo1 antibodies by *in-vitro* experiments (45, 46). This particular antibody specificity belongs mainly to the IgG1 isotype and binds to common epitopes (47).

For the anti-Jo1 antibodies, it was shown that the formation of the major autoepitope is strongly dependent on proper folding of the molecule (46). As a shared risk factor for anti–Jo-1 autoantibody positivity, the HLA–DRB1^*^0301 allele was identified in European as well as African Americans (48). In the Japanese population, HLA–DRB1^*^0405 was associated with the formation of an anti-tRNA antibody response (49).

Another specific antigen is **the transcription intermediary factor-1 gamma** (TIF-1γ). This multi-functional protein with a molecular weight (MW) of 140/155 kilo-Dalton (kDa) is mainly involved in gene transcription (27, 50–53). The TIF-1 family is composed of tripartite motif-containing (TRIM) proteins, which are all implicated in cell proliferation, development, apoptosis, and innate immunity (54). All TIF1 proteins share a C-terminal chromatin reading unit consisting of a plant homeodomain finger and a bromo-domain (BROMO) that is highly conserved among TIF1 family members, but which is not present in any of the other TRIM proteins (55, 56). Of note, while the most common target in anti-TIF1-positive CAM (cancer-associated myositis) is TIF1γ, other proteins of the TIF1 family (TIF1α and β) may also be simultaneously targeted by the immune system (53).

The tripartite containing motif (TRIM) allows these proteins also to function as E3-ligases in the ubiquitination pathway to control protein degradation, localization, and function. In this context, it is interesting to mention that TIF1-γ is involved in the regulation of TGF-β signaling via mono-ubiquitination of SMAD-4 leading to suppression of TGF- β. Thus, by stimulating cell growth and differentiation, TIF1-γ could play a pivotal role in promoting or suppressing malignant cell growth and differentiation (57). The known association of anti-TIF1-γ antibodies with a high risk of cancer development in DM suggest that this link could be not random. Of note, anti-TIF antibodies were only rarely detectable in patients with solid cancer (3.1%) or paraneoplastic rheumatic syndrome (3.3%) without DM (58).

Recently, it was recognized that tumors from paraneoplastic anti-TIF1-γ positive patients showed an increased number of genetic alterations, such as mutations and loss of heterozygosity (LOH) in TIF1 genes (59). Compared with type-matched control tumors from non-myositis patients, TIF1-γ staining was also significantly more intense in tumors as well as muscle tissue from anti-TIF1-γ positive patients. This finding could indicate that the co-occurrence of mutations in peptide regions of TIF1 with high affinity for HLA class I and tumors with high-level TIF1 protein expression may initiate a strong adaptive immune response against neoplastic cells with the mutation. Interestingly, LOH is the most frequent way to lose a mutant allele in human cancer and this is key to tumor immune-editing, since tumor cells with mutations producing a neo-antigen may be eliminated by the immune system and replaced by tumor cells with LOH in that region (without the antigenic mutation) (60, 61). Thus, these modifications can induce an immune response, but also cause an escape of the tumor cell from clearance.

It has been described that DM disease increases toward the equator and strongly associate with latitude. Recently, this observation was confirmed by another study showing that relative prevalence of DM and frequency of anti-TIF1-γ autoantibodies were found to be significantly negatively associated with latitude in adult myositis. Furthermore, HLA alleles HLA-DRB1^*^ 07:01 and HLA-DQB1^*^02 were strongly associated with the DM-specific autoantibodies anti-Mi-2 as well as anti-TIF1-γ (62).

The component of **the nucleosome remodeling deacetylase complex, Mi-2**, is an autoantigen of 240 kDa MW and exists in two isoforms Mi-2α (CHD3) and Mi-2β (CHD4). Mi-2 is responsible for the remodeling of chromatin by de-acetylating histones and plays a role as transcription repressor (63). The role of anti-Mi-2 autoantibodies in the pathogenesis of DM is unclear. However, the autoantigen Mi-2 is found to be up-regulated in the muscle tissue of DM patients. Of note, exposure of keratinocytes to UV radiation has been shown to increase the expression of Mi-2 protein supporting the hypothesis that UV radiation may be associated with the induction of anti-Mi-2 autoantibodies (64). However, a reported increase in the presence of anti-Mi-2 autoantibodies toward the equator was not confirmed by a current study (62, 65). A preferential expression was described in the nucleus of myofibers within fascicles affected by perifascicular atrophy, particularly in the centralized nuclei of small perifascicular muscle fibers expressing markers of regeneration (66). In a mouse model of muscle injury and repair, Mi-2 levels were dramatically and persistently up-regulated during muscle regeneration *in-vivo*. Of note, premature silencing of Mi-2 with RNA interference *in vitro* resulted in accelerated myoblast differentiation. In summary, these results indicate that this protein may play a role in modulating the kinetics of myoblast differentiation. European and American anti–Mi-2 antibody positive DM patients have a common genetic risk factor DRB1^*^0701 (67). Furthermore, HLA-DRB1^*^0302 was identified to be associated with anti–Mi-2 autoantibody positive African American patients (48). Of note, all HLA molecules were found to share a 4–amino-acid sequence motif, which was predicted by comparative homology analyses to have identical 3-dimensional orientations within the peptide-binding groove.

The target antigen of antibodies against **small ubiquitin-like modifier activating enzyme (SAE1/2)** is the SUMO-1 activating enzyme heterodimer with a MW of 40 and 90 kDa, respectively. This antigen is involved in the posttranslational modification of proteins, the so called “sumoylation” (68, 69). Of note, a strong association with the HLA-DRB1^*^04-DQA1^*^03-DQB1^*^03 haplotype has been reported (25). This is another example in IIM, where the association between genotype, serotype and clinical picture suggest a link to the pathogenesis of disease.

Immune reactivity against the **signal recognizing protein hetero-complex (SRP)** is associated with immune-mediated necrotizing myopathy (IMNM). This 72/52 kDa antigen is expressed in the cytoplasm and responsible for transport proteins to the endoplasmic reticulum. SRP consists of six polypeptides (including SRP19 and MIM 182175) as well as seven SL-RNA molecules with partial homology to Alu-DNA (70, 71). Although antibodies against SRP can target each of the different SRP components, a signal peptide-binding 54 kDa subunit (SRP54) represents a major epitope recognized by almost every sera and is, therefore, preferentially used in immunoassays (72–76). Although it is not clear, how the antibodies can interact with the SRP1/2 autoantigen, it was shown that anti-SRP antibodies can inhibit the translocation of secretory proteins into the endoplasmic reticulum *in-vitro* and that a passive transfer of IgG from anti-SRP+ patients with IMNM provoked muscle deficiency through a complement-mediated mechanism in mice model. Interestingly, also active immunization with SRP was able to induce an immune response and provoked disease (76, 77). A correlation between anti-SRP54 antibody titers and disease activity was also shown in a longitudinal follow-up study suggesting a pathogenic role of this antibody entity (78). As a genetic risk factor, HLA- DQA1^*^0102 was identified in anti–SRP autoantibody positive African American patients (48).

The **melanoma differentiation antigen 5 (MDA-5**, synonym CADM-140) with a MW of 140 kDa belongs to the family of RIG-I-like receptors of adhesion molecules and represents a resistance factor against double stranded RNA viruses (49, 79). The MDA-5 molecule plays an important role in the regulation of the immune response by the innate system. In this context, it was shown that MDA-5 bind virus particles, e.g., picornaviruses such as coxsackievirus, and induce an antiviral responses by producing type-I interferons and tumor necrosis factor (80). It was also reported that hyperferritinemia could be a marker for rapidly progressive ILD in anti–MDA-5 antibody positive DM patients (81, 82). In this context many cytokines regulate the ferritin synthesis, including IL-1β, IL-18, TNF, IFN-γ, and IL-6 and several of these cytokines are considered to be involved in IIM pathogenesis. HLA–DRB1^*^0101/^*^0405 was found to be associated with susceptibility to anti–MDA-5 antibody positive DM in the Japanese population (49). Interestingly the same alleles are well-known to play a role in the susceptibility to autoantibody induction in rheumatoid arthritis (83).

Autoantibodies against the **nuclear protein 2 (NXP-2)**, also known as MJ or p140 antibodies, are directed against a nuclear matrix protein complex named NXP2/MORC3, which is involved in regulation of p53-induced cell senescence in the context of oncogenic signals (23). NXP-2 is associated with the small ubiquitin modifier SUMO-2 and represses its expression (84).

The **enzyme 3-hydroxy-3-methylglutaryl-CoA reductase (HMGCR)** was recently identified as a target of autoantibodies induced under treatment with statins. The antigen is expressed in the ER membrane and has a MW of 200/100 kDa. HMGCR catalyzes the conversion of HMG-CoA to mevalonic acid, which is an important step in cholesterol biosynthesis. So far, reports about HMGCR in myositis are rare, however in animal studies it was demonstrated that loss of HMGCR function disrupts vascular stability during developmental processes (4).

## Diagnostic Testing for Autoantibodies in IIM

To assess disease activity and as indicators for muscle injury in IIM, basic laboratory diagnostics are used, such as measurements of levels of inflammatory markers as well as serum activity and/or concentration of muscle specific proteins including creatine phosphokinase (CPK) or myoglobin. However, these markers are unspecific and not helpful to distinguish between the different forms of inflammatory muscle damage. In contrast, the reactivity of the described autoantibodies does not sufficiently correlate with disease activity in IIM, but can rather serve for stratification of the disease process and outcome (85). This section focuses on helpful information with respect to routine procedures of autoantibody detection and the recommended diagnostic approaches, since it is of enormous importance to know these basics for appropriate interpretation of results.

Serum is usually used for the detection of autoantibodies, and test procedures are performed according to the manufacturer's validation. From a preanalytical point of view, time point of venepuncture and fasting status of the patient are not relevant. However, hemolytic, lipemic or contaminated blood samples should not be used, since the released proteins and proteinases can interfere with the immunologic method of detection. Especially in case of a prolonged transportation of the blood sample, serum should be separated in advance by centrifugation at 1,300 g. Subsequently, the serum samples can be stored at 2–8°C for up-to 2 weeks before analysis. A longer period requires freezing at minus 20°C, which presumably allows conservation of the autoantibody reactivity for years (86, 87). Furthermore, some treatment procedures including administration of strong immunosuppressive drugs such as B-cell directed therapies as well as plasmapheresis or administration of intravenous immunoglobulins can influence the result of detection by decreasing the concentration of the autoantibodies.

From the methodical point of few, detection of autoantibodies in IIM is not well-standardized and no international reference samples are available so far. In general, immunoprecipitation of radio-labeled proteins or RNA molecules is still considered to represent the “gold standard” (68). However, due to the time-consuming procedure, high amount of required antigens and low sensitivity, this method is not a routine procedure in most clinical laboratories. Therefore, for the detection of IIM specific and/or associated autoantibodies several, more efficient approaches such as immunofluorescence, ELISA and western blotting are widely used (88–90). In general, immunofluorescence requires large experience to interpret patterns and may not be sensitive enough to detect all MSAs/MAA. On the other hand, ELISA methods using recombinant antigens or immunoblotting with denaturated antigens can probably not detect antibodies to certain conformational epitopes. Line-blot assays allow a qualitative detection of many antibodies in one run. In contrast, and in accordance to the manufacturer specification only a limited number of MSAs/MAAs can be detected by commercial available ELISAs so far.

**Figure 1:**
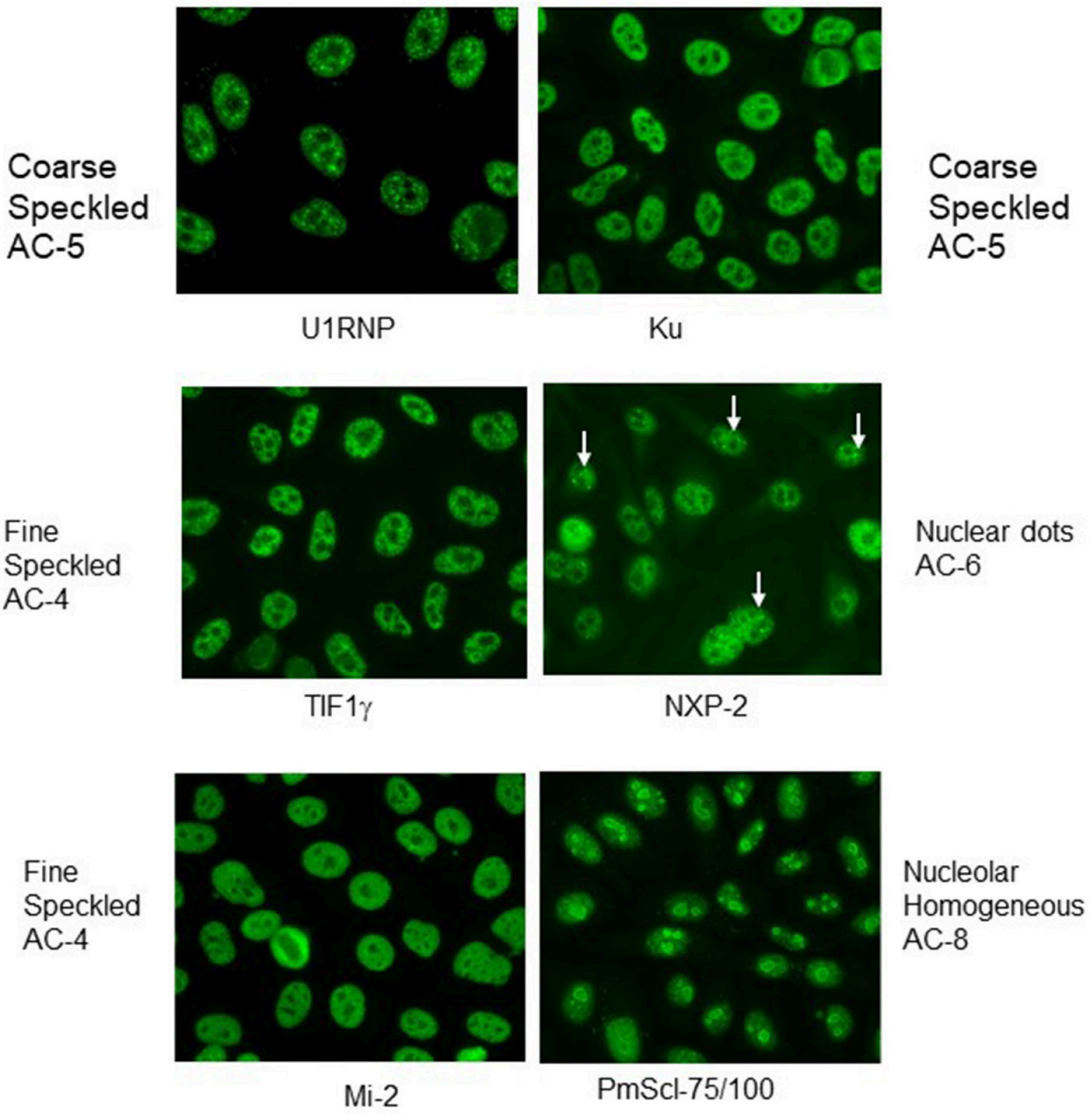
Indirect immunofluorescence with typical nuclear patterns of MSA and MAA. Immunofluorescence patterns are indicated with the terminology of the International Consensus on ANA Patterns (ICAP).

Indirect immunofluorescence (IIF) on HEp-2 cells, a human epithelioma cell line, is commonly be used for detection of anti-nuclear antibodies (ANA), but also enables detection of antibodies against cytoplasmatic antigens. This method allows a screening for a wide range of autoantibodies especially in connective tissue disorders by describing the staining pattern (e.g., nuclear, nucleolar, cytoplamatic), as well as the reactivity titer starting with a serum dilution of usually 1:80. Although no agreement has still been reached on the interpretation and reporting of the ANA titres, results can be considered as weak positive in antibody titer of ≥1:160 and as strong positive in a titres of ≥1:640. In this context, it is important to mention that measured titers often do not correspond with the significance of the results. In other words, a low reactivity should not be considered as irrelevant. Furthermore, it is also important to pay attention to the described pattern of immunofluorescence in the nucleus, but also the cytoplasm. It is helpful to know that IIF on HEp-2 cells shows characteristic patterns of some MSA or MAA antibodies such as those against PM/Scl with prominent homogeneous staining of the nucleous, U1-RNP with coarse speckled staining of the nucleus, Jo-1 or SRP with fine speckled stainings of the cytoplasm (Figure 1), but is not sufficiently accurate to be used as the only screening tool for myositis antibodies.

**Figure 2:**
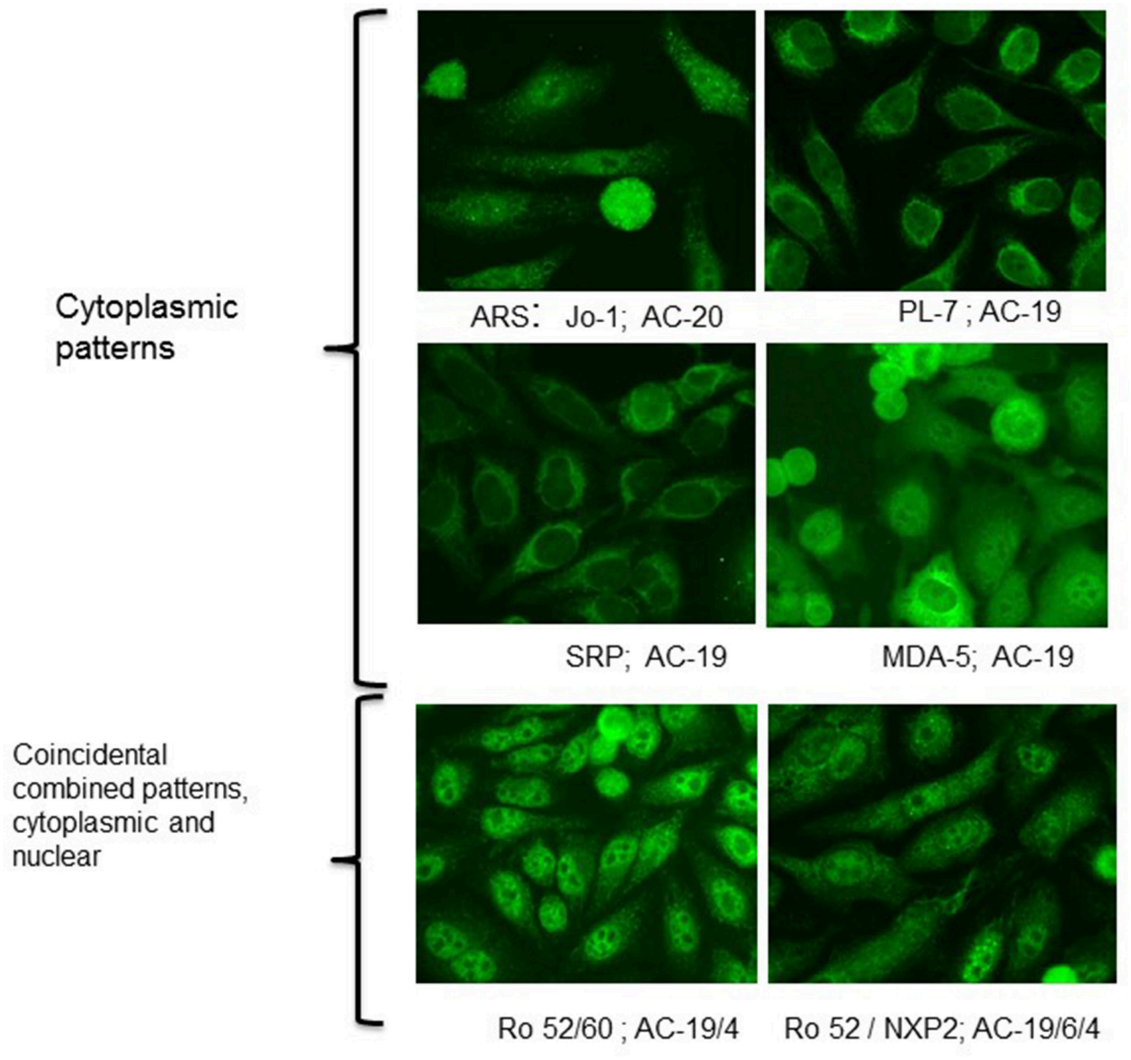
Indirect immunofluorescence with typical cytoplasmic patterns of MSA and MAA. Immunofluorescence patterns are indicated with the terminology of the International Consensus on ANA Patterns (ICAP). AC-19-cytoplasmic dense fine speckled, AC-20-cytoplasmic fine speckled.

Although most of the targeted antigens in IMM are expressed within HEp-2 cells, their detection by IIF is clearly limited. The reasons for frequent false negative results is diverse including e.g., low expression level of the antigen or low affinity of the antibodies causing a weak staining signal. Furthermore, the intracellular distribution of relevant antigens in IIM is usually diffuse generating an unspecific staining pattern. Thus, week positive results are often not recognized, as it frequently occurs even with anti-tRNA synthetase antibodies. Nevertheless, IIF on HEp-2 cells is an important diagnostic procedure. Table 1 summarizes frequent staining patterns in conjunction with the respective antibody and, Figures 1, 2 show representative IIF staining patterns indicating the respective nomenclature for the most frequent MSAs and MAAs by International Consensus on Autoantibody Patterns (ICAP).

**Figure 3:**
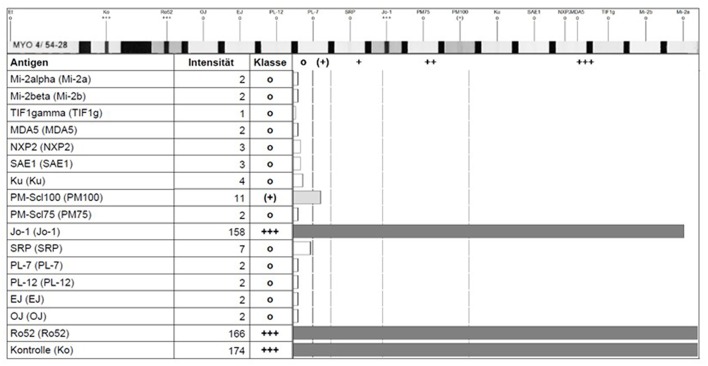
Detection of anti-Jo-1 and anti Ro-52 antibody reactivity in a line-blot assay showing the reaction intensity by a scan-software of the fabricant (EUROLINE, Euroimmune, Germany).

For the detection of classical and new myositis antibodies, multi-analyte line blot assays and ELISA are the current routine methods of choice. In contrast to IIF, commercial ELISAs and line-blot assays use purified or recombinant expressed antigens for detection of antibodies. These methods allow the targeted detection or confirmation of respective antibodies in serum samples, which are usually diluted 1:100. In contrast to semi-quantitative results provided by line-blot assays (with negative, weak or strong positive signals, see Figure 3), ELISA based methods allow a better quantification of antibody reactivity, especially if international standards will be available. However, the majority of ELISA methods published hitherto to detect novel myositis antibodies are in-house made and most of the commercial ELISAs only report negative or positive results by using a cut-off level of reactivity defined by the providing manufacturer. A multiplex-approach for detection of several antibodies in one immunoassay has also limitations and pitfalls due to the difficult optimization of the cut-off for all investigated antibodies. Therefore, it is always important to check the plausibility of the obtained results, not only in cases of weak or borderline reactivity, but also in case of discordance between the different immunoassays (e.g., negative ANA staining pattern but positive anti-PM/Scl antibodies in ELISA or line-asays). On the solid phase of an immunoassay, the antigen structure can be altered yielding a false positive antibody reactivity, which is not directed against the native antigen. Therefore, it is important, that involved diagnostic laboratories are be informed about the clinical suspected diagnosis and provide high quality assays proven by regular internal and external quality controls.

The major advantage of ELISA and line blot test assays is the option of automation test fastness. In this context, the development of commercial quantitative immunoassays including ELISA, chemiluminescence and immunofluorometry has facilitated the use at large scale in routine laboratories and provided interesting information on correlation of clinical and serological findings in IIM.

## Correlation of MSA With Clinical Findings

In addition to inflammatory lesions of skeletal muscles, involvement of other organs such as skin, joint, lung and heart is frequent in IIM. Extra-muscular involvement, especially interstitial lung disease (ILD) and underlying malignancy in cancer-associated myositis (CAM) are the two dominating factors contributing to increased mortality in IIM-patients (91, 92). This section gives an overview on the association of MSA with clinical findings and their diagnostic performance. The incidence of most of the so far identified autoantibody activities of European patients is in agreement with similar studies of Japanese and American patients (7).

## Antibodies Against t-RNA Synthetases

Patients with antibodies against t-RNA synthetases are prone to develop the so called anti-synthetase syndrome (ASS) characterized by myopathy, interstitial lung disease (ILD), non-erosive arthritis, fever, Raynaud's phenomenon and mechanic's hands. Since not all symptoms are present at disease onset, ASS should be carefully considered in patients presenting with isolated arthritis, even in those with erosive manifestation and RF as well as ACPA-positivity (93, 94). The presence of anti-synthetase antibodies can be suspected if a characteristic cytoplasmic pattern on HEp2 cells is evident (Figure 1). However, confirmation is needed using ELISA, immunoblot or line-assays with the isolated antigens. Depending on the diagnostic method anti-Jo-1 antibodies are the most frequent autoantibodies in IIM, they can be detected in 20–30% of patients (7, 95). Titers of Jo-1 antibodies were shown to correlate with disease activity in adults (95). In muscle biopsies of anti-Jo-1 positive myositis patients a specific histologic pattern with peri-fascicular necrosis has been described (96).

The CT- and histomorphologic pattern of ILD in anti-synthetase syndrome can vary between non-specific interstitial and organizing pneumonia (97). Antibodies against PL-7 and PL-12 are positive in up to 5% of IIM patients. Clinically they are associated with less muscle involvement but with a higher proportion of ILD, which might have an acute onset (98). Furthermore, pericarditis was observed in up to 50% of anti-PL7 positive patients (99). Antibodies against OJ, EJ, KS, Zo, and Ha are rare and only present in 1–3% of patients with ARS. The coincidence of anti-Ro52/TRIM21with anti-ASS antibodies was described to be associated with severe myositis and arthropathy as well as with an increased risk of cancer (7, 99, 100). The onset of ILD and myositis, as leading symptoms in ASS, can be subsequently or in parallel, while the course of ILD must not necessarily be progressive.

### DM-Specific Antibodies

**Anti-Mi-2 antibodies** have a high specificity for both adult and juvenile DM (JDM). Mi-2-antibodies produce a fine speckled nuclear pattern in IIF on HEp-2 cells (AC-4 of the ICAP nomenclature) and are detectable in approximately 30% in adult and 10% in juvenile DM (JDM) patients, respectively. Patients with positive anti-Mi-2 antibodies have usually a milder myopathy, lower risk of interstitial lung disease (ILD) and malignancy (101). Skin manifestations, such as Gottron's sign and heliotrope rash, as well, rashes in neck (V rash) and upperback (shawl rash) or cuticular overgrowth are typical. Patients respond well to steroid therapy and have a good prognosis. Furthermore, levels of Mi-2 antibodies were shown to correlate with clinical response to B-cell depletion therapy (102). Although DM with antibodies against Mi2-β can be associated with neoplasia (e.g., colon or mama-carcinoma), anti-Mi-2 antibodies are in general associated with a lower risk of paraneoplastic myositis and hence considered to be a good prognostic factor.

**Anti-TIF1γ** **(anti-155/140) antibodies** are detectable in 13–21% of patients with tumor associated adult DM and in approximately 30% of severe juvenile DM patients (23, 27). In fact, these antibodies are the most frequent marker in juvenile IIM (JIIM) and are primarily associated with JDM. They were originally found almost exclusively in JDM, in about of 23–29% of cases using immunoprecipitation and immunoblotting. Interestingly, in recent studies including a total of 374 cases with JIIM, 131 cases (35%) were found to be positive for these antibodies. In detail, the antibodies were detectable in 38% of patients with JDM (123/320) and in 26% of patients with an overlap syndrome with myositis (8/31) (103, 104). TIF-γ-antibodies are known to be very frequently associated with malignancy in adults with a specificity of 89%, sensitivity of 78%, and positive and negative predictive values of 58 and 95%, respectively (105). In contrast to adults, there is no paraneoplastic association in JIIM (106). Skin manifestations are characterized by a usually slowly progressive onset, but are more extensive than in other JDM groups. In fact, skin ulcerations and lipodystrophy were reported to be particularly associated with these antibodies. However, this was not reported uniformly, since recent studies did not observe ulceration or V-rashes significantly more frequent in this group than in others (104). Of note, it has been shown that levels of TIF-γ-antibodies correlate with response to B-cell depletion therapy in pediatric patients (102).

**Anti-NXP2 antibodies (MJ or p140)** cause a fine speckled ANA pattern on IIF, but this pattern is often misinterpreted as variable nuclear dots (Figure 1). These antibodies have been originally described in children with JDM in about 25% of cases (107, 108). In a cohort of 436 patients with JIIM, their prevalence was reported to be approximately 21% in JDM, 9% in JPM and 15% in overlap syndromes (juvenile connective tissue myositis, JCTM) (104). Subsequently, anti-NXP2 antibodies have been also detected in approximately 30% of patients with DM and 8% with PM in a cohort of 58 adult Italian patients (109), but in only 1.6% of 507 adult Japanese patients (110). In the Italian cohort, a good response to therapy was reported for muscle involvement (109). In juvenile DM, the autoantibodies directed against NXP-2 are frequently associated with calcinosis and ischemic muscle involvement in up to 60% of cases (111). In adults, a possible association was reported with malignancies such as mamma-, uterus- and pancreas-carcinomas.

**Figure 4:**
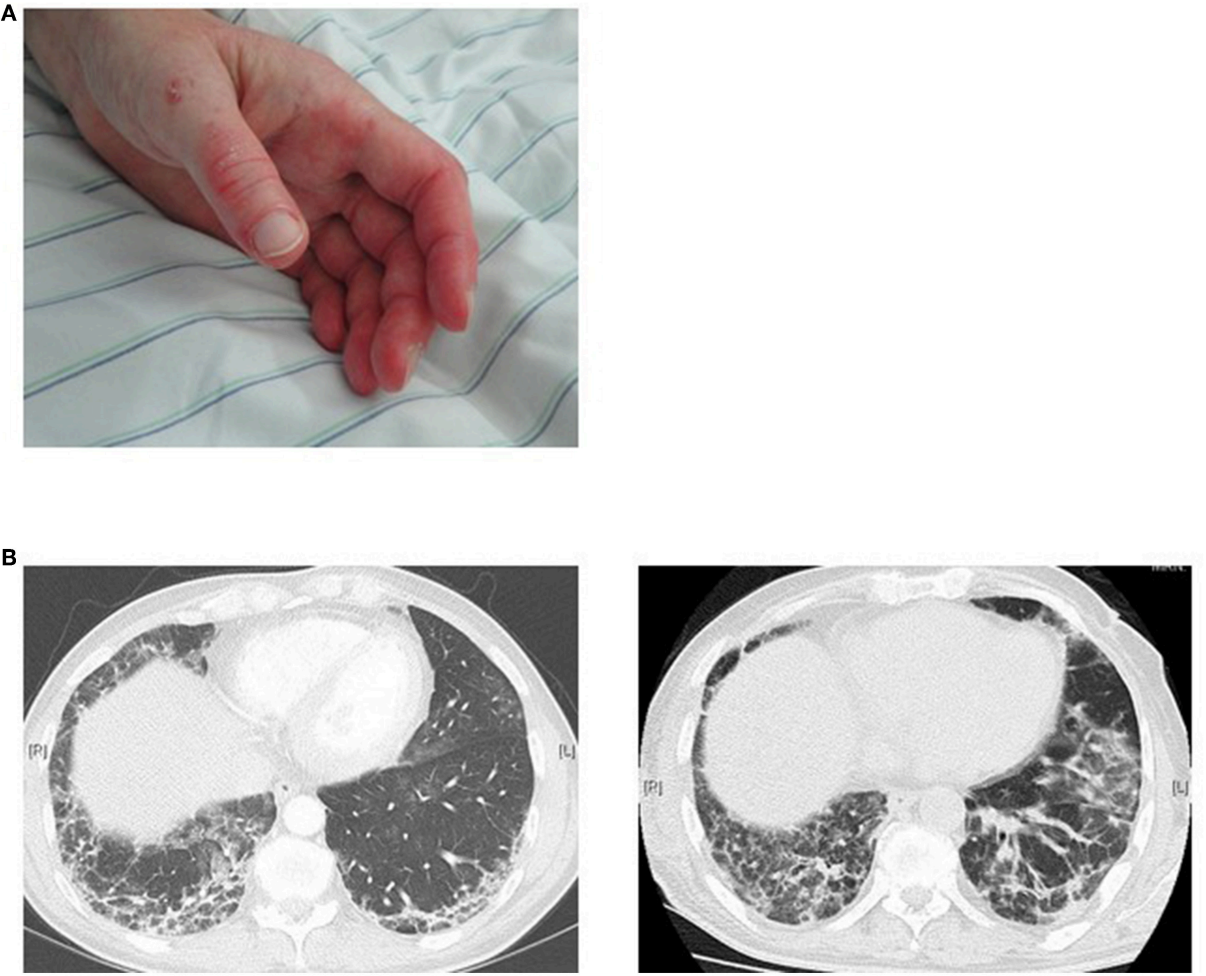
MDA5 positive patient with an amyopathic dermatomyositis **(A)** maculopapular palmar rash and hyperkeratosis, **(B)** rapid progression of ILD manifestations within 5 weeks.

The ANA pattern of **anti-MDA5 antibodies** on HEp-2 cells in IIF is usually negative. These antibodies were identified for the first time in East-Asian populations. They are more frequently observed in adult DM and were reported in a prevalence of 19–35% (19, 81). In a study with 285 patients with JDM (112), anti-MDA5 antibodies were detectable in 7.4% of patients (21/285) and a recent review reports a prevalence of 0–13% in Europe and USA (113). The typical clinical manifestations of adult IIM patients with anti-MDA5 antibodies were amyopathic myositis with rapidly progressive interstitial lung disease (ILD), which in turn determines a high mortality rate in these patients (Figure 4). However, ILD must not be rapidly progressive in every case. In contrast to Asian patients, Caucasian patients often show skin ulcerations and painful palmar papules (114, 115). Furthermore, differences were also reported for Asian compared to Caucasian JDM associated with MDA-5 antibodies. In this context, Japanese patients showed a pulmonary involvement in nearly 50% of patients, whereas the incidence of ILD was much lower in Caucasians. JDM patients with anti-MDA5 antibodies show frequent skin as well as oral ulceration, but no calcinosis and had less severe muscle weakness compared other JDM subtypes. Of note, ILD was found in 4 of 12 cases (19%) on chest X-ray. None of these anti-MDA5 antibodies positive ILD patients was positive for anti-synthetase antibodies suggesting that anti-MDA5 antibodies represent an own subtype of IIM. This study also revealed that more patients with anti-MDA5-antibodies were in remission after 2 years compared to patients without these antibodies. In the follow-up, patients in remission showed a decline in the titer of MDA5 antibodies. These results resemble those of prior studies in which antibodies were shown to disappear during disease remission (20).

The **anti-SAE-antibodies** were originally described in adults with DM exhibiting an amyopathic onset with skin manifestations, but who may develop myositis several months later. Lung involvement was infrequent reported. The antibodies were detected in approximately 7–8% of adult Caucasian patients with DM (25, 116). In a study with 110 Japanese patients with DM including 13 with JDM, only 2 patients with DM (1,8%) were shown to have SAE-antibodies (117). In a large study with 436 patients with JIIM, only one patient with JDM was positive for **anti-SAE-antibodies** (118). Therefore, the presence of SAE-antibodies in patients with JIIM seems to be extremely rare.

### Immune Mediated Necrotizing Myositis (IMNM)

**Anti-SRP-Antibodies** were detected in about 3 to 7% of adults with IIM (73, 119). In JIIM, these antibodies are even more infrequently observed in only 1.6% of patients exclusively with juvenile polymyositis (JPM) (103, 120). The presence of SRP-antibodies can be suspected by detection of a cytoplasmic staining pattern in ANA IIF (Figure 1). Adults with anti-SRP-antibodies develop typically an acute necrotizing myopathy with prominent muscle impairment without skin manifestations. Compared to other myositis forms, a satisfactory response to medicament treatment is difficult to achieve. Pulmonary involvement, Raynaud-symptoms, arthritis or overlap syndromes are infrequent. Similar to adults, juvenile patients have very high levels of CPK as well as often cardiac involvement detectable e.g., by ECG or echocardiography. Of note, adult patients show frequently Raynaud-symptoms, dysphonia and dyspnea under exertion (107). Compared to other forms of JIIM, onset of symptoms is late in patients with anti-SRP-antibodies. However, two unusual cases have been published recently with onset in the first decade of life showing muscular dystrophy and a low degree of inflammation in muscle biopsy (107). The antibody titer against SRP seems to correlate with clinical activity as well as with levels of CPK in adults as well as in JIMM and can be used for monitoring of therapy (78, 121).

Antibodies against the enzyme **3-hydroxy-3-methylglutaryl-CoA reductase** (HMGCR) have been identified initially in patients with immune mediated necrotizing myositis (IMNM) in association with statin treatment. Overall, autoantibodies against HMGCR are detectable in 6–7% of patients with IIM (28–30). Myalgias, as a common side effects under statins, are not associated with this form of myositis and positive HMGCR-antibodies. In fact, only a minority of patients under statin treatment develops myopathy with anti-HMGCR-antibodies (122). Furthermore, even patients without statin treatment can develop necrotizing myositis with positive anti-HMGCR-antibodies (123). Results of a multicenter study show that the majority of patients with anti-HMGCR antibodies have IMNM. In this study, the prevalence of anti-HMGCR antibodies in different subpopulations of IMNM exposed to statins was approximately 70%, and even 75% in patients above the age of 50 years. However, approximately 45% of INMN patients had no exposure to statins and in 5% of cases did not show muscle necrosis (124). In contrast to non-immune statin myopathy, which resolves after stopping statin therapy, patients with anti-HMGCR antibodies have a persistent autoimmune response despite cancelation of treatment with the inducing agent. Similar to SRP antibodies, the titer of anti-HMGCR antibodies correlates with the clinical activity of necrotizing myositis. However, in contrast to SRP antibodies it is not known, whether antibody titres can normalize under effective therapy (125–127). Of note, these antibodies have also been detected in children after statin therapy recently, however, the experience with these cases is very limited (128, 129).

The detection of anti-HMGCR antibodies was performed by only few laboratories using immunoprecipitation so far, since no standardized commercial assay was available. An introduction of other methods such as ELISA or chemiluminescence for the detection of anti-HMGCR antibodies could make this test widely available to facilitate the diagnostic possibilities (130). However, since such immunoassays can yield false positive results due to detection of low avidity antibodies, a confirmatory analysis by immunoprecipitation can be recommended (131). Interestingly, usage of rat hepatocytes in indirect immunofluorescence was proposed (132). However, although some laboratories have adopted this strategy, there are not yet available data from an extended use of this approach (133). Of note, in a French cohort study HMGCR positive IMNM-patients were found to be at higher risk for malignancy (134).

## Autoantibodies in DM and the Risk of Cancer

A high risk of cancer development is well-described and has led to the sub-categorization of cancer associated myositis (CAM) (135). The overall risk of cancer in myositis is significantly higher than in the age-matched population and approximately 10% of IIM are associated with malignancies (134–138). In DM, the risk is especially increased in the first 5 years after diagnosis (139–144). The most common cancers among the reported cases associated with DM are nasopharyngeal carcinoma and adenocarcinoma of the ovary, lung, pancreas, stomach and colon (139–145). Many studies have claimed the presence or absence of certain antibodies as markers for the risk of cancer in myositis. The autoantibody profile is considered a useful tool to identify patients at risk for CAM (32). In this context, the best established antibodies are directed against TIF1-γ with a high sensitivity and specificity for cancer associated DM in adult patients (27, 52, 53, 105, 146, 147), but not in JDM (50). Furthermore, also NXP-2 positivity in DM patients has been identified as a risk factor for malignancy (148). The association of anti-synthetase and anti-HMGCR antibodies with cancer is less clear and needs further confirmation. To exclude an associated malignancy in clinical practice repetitive examinations can be recommended in DM patients at risk, whereas clear guidelines on the frequency and extension of such diagnostic procedures are missing so far.

## Summary

Autoantibodies in IIM are very important diagnostic and prognostic markers, which can help to facilitate our approach to these rare and divers diseases. They correlate closely with the clinical manifestation of disease and allow stratification of patients. However, a laboratory result provides always only a piece of the diagnostic puzzle and should always be questioned if not plausible. For this purpose, a better knowledge on the limitations of the laboratory procedures is necessary and tests should only be performed if indicated according to the clinical picture. To allow comparison and improve reproducibility, the existing assays require uniform standards on an international level, and optimized methods for a broader distribution. If these issues can be solved, antibodies in IIM will play a more prominent role in the classification of disease. Another interesting and important tasks for the future will be to investigate the treatment response in IIM patients according to their antibody profile in more detail. This includes the question, whether different antibodies correlate with disease activity, but can be also used for an individualized approach to predict the response and outcome. Finally, the interaction of the antibodies with the targeted antigens is of major interest especially for the IIM specific entities. A deeper understanding on the mechanisms behind the induction of the respective immune response as well as on the potential role of the targeted antigens in IIM can improve our insight into the pathogenesis of disease.

## Author Contributions

BS reviewed published articles and contributed to the manuscript on antigen-antibody systems. US reviewed published articles and contributed to the manuscript on clinical aspects. J-BG-G reviewed published articles and contributed to the manuscript on laboratory diagnostic aspects. EF reviewed published articles and contributed to all parts of the manuscript.

### Conflict of Interest Statement

The authors declare that the research was conducted in the absence of any commercial or financial relationships that could be construed as a potential conflict of interest.
